# Increased mitochondrial DNA damage and down-regulation of DNA repair enzymes in aged rodent retinal pigment epithelium and choroid

**Published:** 2008-04-04

**Authors:** Ai Ling Wang, Thomas J. Lukas, Ming Yuan, Arthur H. Neufeld

**Affiliations:** Forsythe Laboratory for the Investigation of the Aging Retina, Department of Ophthalmology, Northwestern University School of Medicine, Chicago, IL

## Abstract

**Purpose:**

In the central nervous system (CNS), increased mitochondrial DNA (mtDNA) damage is associated with aging and may underlie, contribute to, or increase the susceptibility to neurodegenerative diseases. Because of the focus on the retinal pigment epithelium (RPE) and choroid as tissue relevant to age-related macular degeneration (AMD), we examined young and aged RPE and choroid, harvested from rodent eyes, for DNA damage and for changes in selected DNA repair enzymes.

**Methods:**

Immunohistochemical labeling and quantitative ELISA for the oxidative DNA damage marker, 8-hydroxy-2’-deoxy-guanosine (8-OHdG), were measured in young and aged rodent RPE and choroid. mtDNA and nuclear DNA (nDNA) damage was determined by quantitative polymerase chain reaction (PCR) by comparing the relative amplification of small and large DNA fragments. Expression of several DNA repair enzymes was measured using real-time quantitative reverse transcription -PCR (qRT–PCR) and immunoblot.

**Results:**

Immunohistochemical labeling for 8-OHdG increased in aged rodent RPE and choroid. Quantitative ELISA confirmed increased levels of 8-OHdG. Measurements of nDNA and mtDNA lesions indicated that DNA damage is primarily in mtDNA in aged RPE and choroid. Using qRT–PCR, we found that gene expression of DNA repair enzymes, 8-oxoguanine-DNA glycosylase 1 (OGG1), mutY homolog (MYH), and thymine DNA glycosylase were decreased in an age-dependent pattern in RPE and choroid. However, endonuclease III homolog 1 was not significantly changed in aged RPE and choroid. Using immunoblots, we found that protein levels of OGG1 and MYH were decreased in aged RPE and choroid.

**Conclusions:**

Our results show that there is increased mtDNA damage in aged RPE and choroid, which is likely due to decreased DNA repair capability. mtDNA damage in the RPE and choroid may be a susceptibility factor that underlies the development of AMD.

## Introduction

Age is a risk factor for adult human neurodegenerative diseases [1]. However, the cellular and molecular explanations for that clinical statement are not generally known for most tissues [2]. Older individuals may have underlying changes in specific neural tissues that contribute to the cause as well as progression of age-related disease processes. These age-related changes may increase the susceptibility of the tissues to the causative disease processes. Thus, these changes are susceptibility factors and may be manifested clinically as risk factors.

There are characteristic accumulations of molecular damage at the subcellular level during aging [3]. A susceptibility factor underlying age-related neurodegenerations is the increased presence of damaged DNA with age [4,5]. Accumulated damage to DNA is a threat to proper cellular functions, responses and survival [6]. Although there are a variety of DNA lesions that increase with age, oxidation-induced mitochondrial DNA (mtDNA) damage is implicated in contributing to many age-related disease states [7].

The detrimental effects of oxidative DNA damage are readily observed in the central nervous system (CNS) [8]. This damage is caused by reactive oxygen species (ROS), reactive nitrogen species, and other free radicals produced by metabolic activity. Oxidative damage accumulates in the DNA of CNS neurons over time, especially in the mtDNA, and may contribute to the pathogenesis or progression of neurologic disorders, such as Parkinson disease, amyotrophic lateral sclerosis, and Alzheimer disease [9]. Age-related diseases of the eye, such as glaucoma and age-related macular degeneration (AMD), may also be associated with mtDNA damage.

Our laboratory is identifying, at the molecular level, susceptibility factors for AMD. AMD is a devastating disease associated with aging that destroys the sharp, central vision that is needed for seeing objects clearly and for common daily tasks such as reading and driving. Age is the greatest risk factor for AMD. Increased levels of oxidative DNA damage in the retinal pigment epithelium (RPE) and choroid with age may contribute to the pathogenesis and progression of AMD [10,11].

Age-related damage to DNA in the RPE and choroid has not been characterized in vivo. The purpose of the work presented here was to determine if there is increased damaged mtDNA with age in the RPE and choroid. We have characterized and compared the state of the mtDNA in the RPE and choroid from young and old rodents by assaying rodent tissue for oxidative DNA damage and lesions in mtDNA and nuclear DNA (nDNA) by quantitative PCR. We also evaluated the transcriptional and translational levels of selected oxidative DNA damage repair enzymes.

## Methods

### Animal models

Six male C57BL/6 mice in each group (5 month and 22 month) and twelve male Brown-Norway rats in each group (5 month and 26 month) were provided by National Institute on Aging. Animal were housed conventionally, under ambient conditions (10 h dark, 14 h light) and were fed standard rodent chow and water ad libitum. Six frozen eyes of male C57BL/6 mice in each group (4, 12, 18, and 24 month) and ten frozen eyes of male F344xBN F1 rats in each group (4, 18, 24, and 32 month) were provided by the tissue bank of National Institute on Aging. All experiments were performed in accordance with the United States Public Health Service Policy on Humane Care and Use of Laboratory Animals, the National Institutes of Health Guide for the Care and Use of Laboratory Animals, the ARVO Statement for the Use of Animals in Ophthalmic and Vision Research, and institutional, federal, and state guidelines regarding animal research.

**Table 1:** DNA primers used for DNA amplification and qRT–PCR

**Long fragment of mitochondrial DNA (13.4 kb)**
5′-AAAATCCCCGCAAACAATGACCACCC-3′ Sense
5′-GGCAATTAAGAGTGGGATGGAGCCAA-3′ Antisense
**Short fragment of mtDNA (235 bp)**
5′-CCTCCCATTCATTATCGCCGCCCTTGC-3′ Sense
5′-GTCTGGGTCTCCTAGTAGGTCTGGGAA-3′ Antisense
**Long fragment of nDNA (12.5 kb)**
5′-AGACGG GTGAGACAGCTGCACCTTTTC-3′ Sense
5′-CGAGAGCATCAAGTG CAG GCA TTAGAG-3′ Antisense
**Short fragment of nuclear DNA (195 bp)**
5′-GGTGTACTTGAGCAG AGC GCTATAAAT-3′ Sense
5′-CACTTACCCACGGCAGCTCTCTAC-3′ Antisense

### Immunohistochemistry

Enucleated eyes were fixed in 2% wt/vol paraformaldehyde in 0.01 M phosphate buffered saline (PBS; pH 7.4) at 4 °C overnight. Six live animals were used for each group in all immunohistochemistry experiments. Immunohistochemistry was performed on paraffin sagittal sections of eyes after pigment bleaching for 1:200 dilution 8-hydroxy-2’-deoxy-guanosine (8-OHdG; Oxis, Foster City, CA), using the Vectastain Elite ABC kit (Vector Laboratories, Burlingame, CA) and diaminobenzidine as a substrate. As a negative control, sections were treated in the same manner, except that incubation with primary antibody was omitted. The sections were treated for equal time in DAB reagent and photographed at the same time.

**Figure 1:**
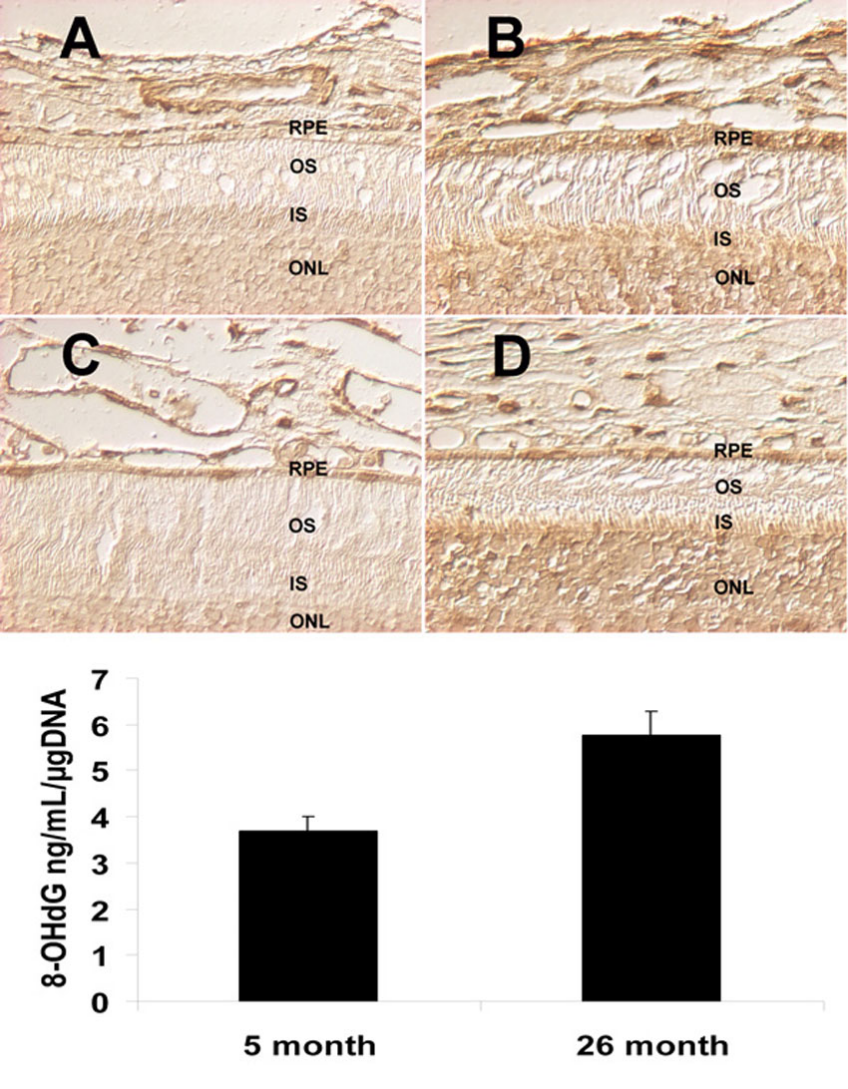
Increased 8-hydroxy-2’-deoxy-guanosine in the aged retinal pigment epithelium and choroid. Immunolabeling for 8-hydroxy-2’-deoxy-guanosine (8-OHdG) of retinal pigment epithelium (RPE) and choroid from from young mice (5 month, **A**), young rats (5 month, **C**) compared to old mice (22 month, **B**) and old rats (26 month, **D**). The labeling intensity of 8-OHdG was higher in the old RPE and choroid from mice (**B**) and rats (**D**), compared to the young RPE and choroid (**A** and **C**). Scale bar equals 20 µm. The oxidative DNA adduct, 8-OHdG, in rat RPE and choroid was quantitatively measured by 8-OHdG ELISA (**E**). There was a significant increase (p=0.007, n=6) in the amount of 8-OHdG in aged RPE and choroid.

### 8-hydroxy-2’-deoxy-guanosine ELISA

Genomic DNA was isolated using a DNeasy Blood & Tissue Kit (Qiagen, Valencia, CA) from six live rat RPE and choroid, following hydrolysis with a four enzyme mixture including DNase I, phosphodiesterases I and II and alkaline phosphatase [12]. Competitive ELISA assays for 8-OHdG were performed according to the manufacture’s protocol (Bioxytech 8-OHdG-EIA kit; Oxis). Briefly, the 8-OHdG antibody and the sample were added to ELISA plate which has been precoated with 8-OHdG. The 8-OHdG in the sample competes with the 8-OHdG bond on the plate for the 8-OHdG antibody bites binding sties. The antibodies that are bound to the 8-OHdG in the sample were washed out of the well, while those that have bound to the 8-OHdG coated on the plate will remain. Following secondary antibody and chromogen, the color reaction was terminated and the absorbance was measured.  Line 100~102: Standard 8-OHdG was assayed over a concentration range of 0.125 to 20 ng/ml in duplicates for each experiment. The average concentration of 8-OHdG per microgram of DNA for each group was calculated for each sample. Sample DNA assays were performed in duplicate. Standard 8-OHdG was assayed over a concentration range of 0.125 to 20 ng/ml in duplicates for each experiment the average concentration of 8-OHdG per microgram of DNA for each group was calculated for each sample. Controls without added DNA and appropriate blanks were also incorporated into experiments.

### Long extension-polymerase chain reaction

Long extension-polymerase chain reaction (LX-PCR) was performed as previously described [13]. Genomic DNA in RPE and choroid from five frozen rat eyes in each group was isolated with DNeasy Blood & Tissue Kit (Qiagen). The quantitation of the purified genomic DNA, as well as of PCR products, was performed fluorometrically using the PicoGreen dsDNA reagent (Invitrogen, Carlsbad, CA). LX-PCR was performed with the GeneAmp LX-PCR was performed with the GeneAmp XL PCR system (Applied Biosystems), using rTth DNA Polymerase XL enzyme which is designed to amplify target DNA sequences up to about 40 kb. The amounts of primers were 20 pmol, and the Mg^2+^ concentration was 1.1 mM. The four pairs of PCR primers employed in this study are given in Table 1. All the protocols were initiated by a hot start (75 °C, 2 min) before addition of rTth enzyme. For amplification of a long fragment of mtDNA, the standard thermocycler program included initial denaturation at 94 °C for 1 min, 18 cycles of 94 °C for 15 s, 65 °C for 12 min, with final extension at 72 °C for 10 min. Amplification of a short mtDNA fragment (235 bp) was done with the same program except the extension temperature was 60 °C. To amplify a long (12.5 kb) nDNA fragment, the thermocycler profile included initial denaturation at 94 °C for 1 min, 28 cycles of 94 °C 15 s, 65 °C 12 min, with final extension at 72 °C for 10 min. A short nDNA fragment (195 bp) was amplified with the same program except the extension temperature was 60 °C.

DNA damage was quantified by comparing the relative efficiency of amplification of large fragments of DNA (13.4 kb from mtDNA and 12.5 kb for nDNA) and normalizing this to the amplification of smaller (235 bp and 195 bp) fragments. The template DNA (5~15 ng) was varied so that PCR products were obtained during the long phase of the PCR amplification.

**Figure 2:**
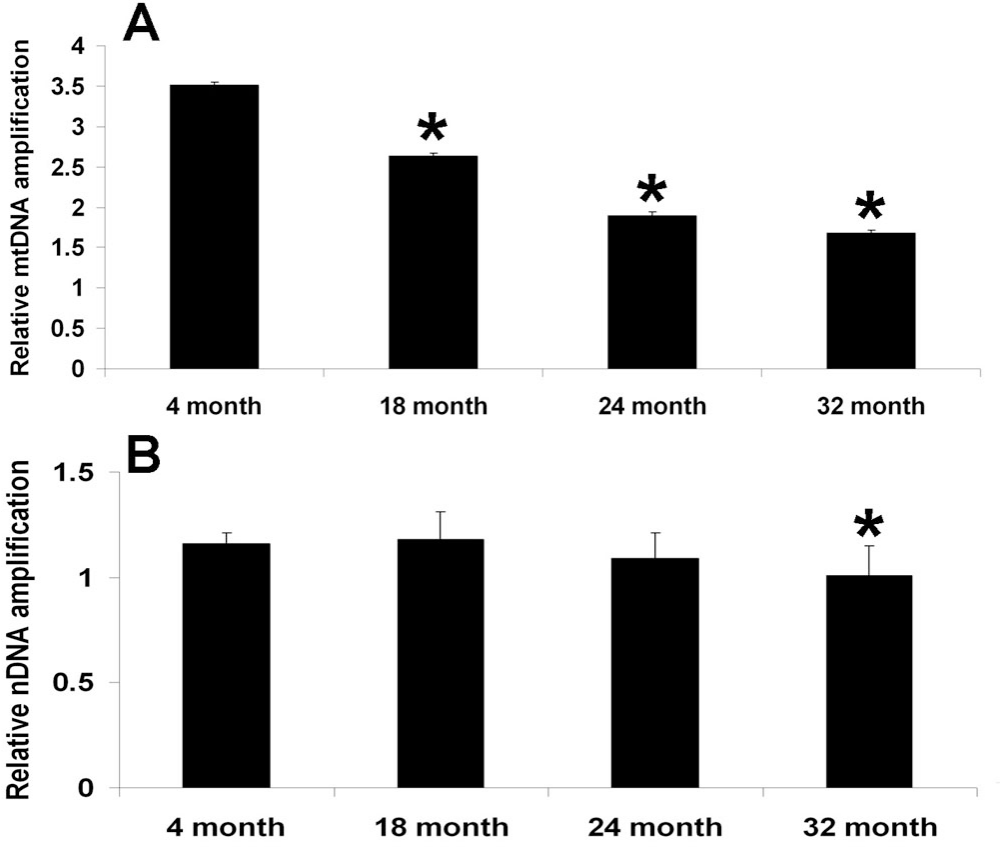
Increased mitochondrial DNA damage compared to nucleic DNA in aged retinal pigment epithelium and choroid. DNA products from the polymerase (rTth) mediated amplification of long fragments of mitochondrial DNA (mtDNA) (13.4 kb) and nuclear DNA (nDNA) (12.5 kb) from retinal pigment epithelium (RPE) and choroid were measured. These data were normalized by the measured levels of the short fragment of mtDNA and nDNA obtained using the sample DNA template sample. As shown in Panel **A**, there was a significant decrease in mtDNA amplification beginning at 18 month (p=0.004, n=5), which decreased progressively at 24 month (p<0.0001, n=5) and at 32 month (p<0.0001, n=5), compared with the 4 month group. As shown in Panel **B**, there was no change in the nDNA amplification at 18 month (p=0.54, n=5) and 24 month (p=0.06, n=5), compared with the 4 month group. At 32 month (p=0.002, n=5), there was a significant decrease in nDNA amplification, compared with the 4 month group

### Amplification of total, non-deleted, and deleted mitochondrial DNA

Genomic DNA in RPE and choroid from five frozen rat eyes in each group was isolated. Three nonoverlapping regions of the mitochondrial genome were amplified by conventional PCR to evaluate the relative proportion of total, non-deleted, and deleted mtDNA in old rats, compared to those in young rats. The three pairs of 20-nucleotide PCR primers (Table 2) employed in this study were similar in GC content and melting temperatures. The total primer pair amplified a segment of mtDNA outside of the 4.8 kb common deletion; the non-deleted primer pair amplified a region within the common deletion; and the deletion primer pair amplified a product only in the presence of the common deletion, which would bring the primer pairs within 500 bp. Thermocycling included one cycle of 94 °C for 5 min, followed by 40 cycles of 94 °C denaturing for 45 s, 55 °C annealing for 45 s, and 72 °C extension for 60 s. A final 72 °C extension for 5 min completed the PCR. Negative controls were included that contained the PCR reagents without template DNA.

To determine the amounts of DNA products obtained in these reactions, we isolated total DNA and used the aforedescribed three primers for amplification of total, non-deleted, and deleted mtDNA in young and aged RPE and choroid. The amount of PCR product was determined fluorometrically using the PicoGreen dsDNA quantitation reagent (Invitrogen).

### Real time reverse transcriptase–polymerase chain reaction for DNA repair enzymes

Total cellular RNA in RPE and choroid from 6 frozen mice eyes was isolated and purified (PicoPure^TM^; Arcturus, Mountain View, CA). Samples of the total starting RNA were analyzed by capillary electrophoresis (Agilent Technologies, Palo Alto, CA) to assess the degree of purification. Real time quantitative RT–PCR (qRT–PCR) was done using the SYBR-Green dye binding method implemented on an Applied Biosystems 7900 genetic analyzer by the Functional Genomics Facility at the University of Chicago. Validated primers for each gene of interest were designed for each target mRNA (Table 3). Optimization of primers and determination of the input cDNA levels were done to ensure appropriate cycle time response. Relative expression was calculated from the differences in cycle time of an internal standard (18s RNA) compared to the target mRNA. All qRT–PCR reactions used 40 ng of cDNA produced as described above and were run with two sets of duplicates from six different animals in each group.

### Immunoblot

RPE and choroid from six rats (4 month and 32 month) were dissected and pooled, lysed in buffer (20 mM HEPES, pH 7.0, 10 mM KCl, 2 mM MgCl_2_, 0.5% Nonidet P-40, 1 mM Na_3_VO_4_, 1 mM PMSF, and 0.15 U ml^–1^ aprotinin) and homogenized. Protein concentrations were determined using the Bradford colorimetric assay. Thirty micrograms of each protein lysate were loaded in each lane in sample buffer (2% SDS, 10% glycerol, 0.001% bromophenol blue, 1% DTT, and 0.05 M Tris-HCl, pH 6.8), separated on 10% SDS–PAGE (Invitrogen), and transferred to a PVDF membrane (Millipore, Temecula, CA). The blots were blocked with 5% nonfat milk in PBS and incubated with specific rabbit polyclonal antibody against 1:200 8-oxoguanine-DNA glycosylase 1 (OGG1), mutY homolog (MYH) (Novus) followed by peroxidase-conjugated goat antirabbit IgG_2a_ (Millipore) and the enhanced chemoluminescence detection system (Amersham Biosciences, Arlington Heights, IL). The differences in expression levels were determined by scanning gels and determining the integrated areas of the bands using Image-J software. Data are expressed as normalized ratios to actin.

**Table 2:** Primers for mtDNA amplification

**Total mtDNA (504 bp)**
5′-CACACTCTCACTCGCATGAA-3′ Sense
5′-TCCTTCCAATCTAGTTGAGG-3′ Antisense
**Non-deleted mtDNA (369 bp)**
5′-ACTCCAACTCCATAATCTCC-3′ Sense
5′-TATTAGTGGGAGGAGTCAAG-3′ Antisense
**Deleted mtDNA (348 bp)**
5′-GGTCTACCAATTGTTGTGAC-3′ Sense
5′-TAGTGAGATAAGGAAGCCTG-3′ Antisense

### Statistical analyses

Data are presented as mean±standard error of the mean (SEM) with statistical differences between groups analyzed by a standard Student two-tailed *t*-test using GraphPad Prism software. A p value of less than 0.05 was considered statistically significant.

## Results

### 8-hydroxy-2’-deoxy-guanosine immunohistochemistry

We compared young and aged RPE and choroid tissues for the presence of 8-OHdG as an indicator of oxidative DNA damage. The labeling intensity in mice and rats was higher in the old RPE and choroid than the young RPE and choroid. Figure 1 shows the immunolabeling of RPE and choroid from young mice (5 month, n=6) and young rats (5 month, n=6) compared to old mice (22 month, n=6) and old rats (26 month, n=6). Although relative intensity differed from cell to cell, RPE and choroid from old eyes has compact labeling, indicating increased oxidative damage to DNA. The specificity of the antibody that we used has been established [14], and we further confirmed the specificity for immunohistochemistry by observing that there was no reduction in labeling following pretreatment with DNAase-free RNAase (data not shown).

**Figure 3:**
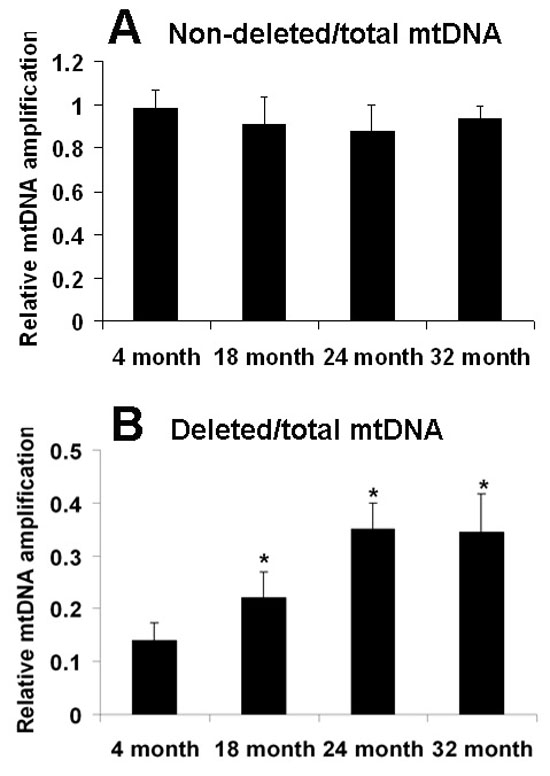
Increased levels of deleted mitochondrial DNA in aged retinal pigment epithelium and choroid. Measurements of levels of the PCR products of non-deleted (**A**, not damaged) and deleted (**B**, damaged) mitochondrial DNA (mtDNA) normalized by total mtDNA normalized by total mtDNA were done using the PicoGreen reagent. Each PCR reaction started with 10 ng of genomic DNA (nuclear and mitochondrial) from rat retinal pigment epithelium (RPE) and choroid as a template. There was no difference in the non-deleted mtDNA at 18 month (p=0.02, n=5), 24 month (p=0.06, n=5), and 32 month (p=0.70, n=5), compared with the 4 month group. However, there was a significant increase in deleted mtDNA at 18 month (p=0.003, n=5), 24 month (p<0.0001, n=5), and 32 month (p<0.0001, n=5), as compared with the 4 month group. Values are the mean±SEM.

### 8-hydroxy-2’-deoxy-guanosine ELISA

We complemented the immunohistochemical results using ELISA. Because greater amounts of DNA were available from rat RPE and choroid tissues, which are substantially larger than mice, we performed ELISA determinations for 8-OHdG on young (5 month) and old (26 month) rat samples. ELISA also provided a quantitate estimate of oxidative DNA damage. In the young group, the level of 8-OHdG was 3.7±0.3 ng/ml/µg DNA. In the old group, the level of 8-OHdG was 5.8±0.5 ng/ml/µg DNA (Figure 1E). The amount of 8-OHdG in DNA was significantly increased in aged RPE and choroid (p=0.007, n=6).

### Long extension quantitative polymerase chain reaction of mitochondrial DNA and nuclear DNA

Cellular DNA is composed of mtDNA and nDNA. In our study of the RPE and choroid, we used quantitative PCR-based measurements to evaluate oxidative damage in nDNA and mtDNA. These methods required only small amounts of genomic DNA and were used to directly compare damage to nDNA and mtDNA in the same sample [13,14]. In postmitotic cells, greater levels of oxidative DNA damage with age occur more commonly in mtDNA than in nDNA [15], which is consistent with our findings.

We used an independent method to determine global DNA damage to both nDNA and mtDNA. The quantitative PCR assay of DNA damage is based on the principle that many kinds of DNA lesions can slow down or block the progression of DNA polymerase [13]. Therefore, equal amounts of DNA in less damaged samples will amplify to a greater extent than samples with more damaged DNA. DNA products from the polymerase (rTth) mediated amplification of long fragments of mtDNA (13.4 kb) and nDNA (12.5 kb) from RPE and choroid of frozen eyes (4, 18, 24, and 32 month) were measured using the PicoGreen reagent that is selective for dsDNA products. These data were normalized by the measured level of the short fragment of mtDNA and nDNA obtained using the same DNA template.

As shown in Figure 2A, there was a significant decrease in mtDNA amplification beginning at 18 month (2.6±0.1; p=0.004, n=5), which progressively decreased at 24 month (1.9±0.1; p<0.0001, n=5) and at 32 month (1.7±0.1; p<0.0001, n=5), compared with the 4 month group (3.5±0.2; n=5). As shown in Figure 2B, there was no change in the relative nDNA amplification comparing the 4 month group (1.16±0.05, n=5) to 18 month group (1.18±0.13, p=0.54, n=5), or to 24 month group (1.09±0.12, p=0.06, n=5). However, the 32 month group (1.01±0.14, p=0.002, n=5) exhibited a significant decrease in nDNA amplification compared to the 4 month group (1.16±0.05, n=5).

**Table 3:** Primers for qRT–PCR

**OGG1**
5′-GCTCGCATTACTGGCATGGT-3′ Sense
5′-GCTGAATGAGTCGAGGTCCAA-3′ Antisense
**MYH**
5′-GGCCGTCGGCTACAAGAAG-3′ Sense
5′-TGGCATATGGCCTCCTAGCT-3′ Antisense
**NTH1**
5′-TGCTCTCCAGCCAGACCAA-3′ Sense
5′-CCCGGAGCCGTTGCA-3′ Antisense
**TDG**
5′-CCTCCGTGGACTCGAAGCT-3′ Sense
5′-TCTGCGTGTGACTGCAAACC-3′ Antisense

### Mitochondrial DNA polymerase chain reaction

Amplification of total, non-deleted (not damaged), and deleted (damaged) PCR products of mtDNA were also done using total genomic DNA from rat RPE and choroid of all groups as a template. The total input of genomic DNA was 10 ng for each PCR reaction. Amplification products representing the total, non-deleted, and deleted mtDNA were detected in young and old rat RPE and choroid (Figure 3). Non-deleted mtDNA remained stable through much of the lifespan at 18 month (0.9±0.02; p=0.02, n=5), 24 month (0.9 ±0.05; p=0.06, n=5), and 32 month (0.9±0.1; p=0.70, n=5), compared with 4 month (1.0 ±0.02, n=5). However, there was a significant increase in deleted mtDNA at 18 month (0.2±0.1) (p=0.003, n=5), at 24 month (0.4±0.05; p<0.0001, n=5) and at 32 month (0.3±0.1; p<0.0001, n=5), compared with the 4 month group (0.1±0.03). These data independently confirm the long extension PCR experiments that demonstrated an increase with age in the level of mtDNA containing deletion products in old RPE and choroid tissue.

### Real time reverse transcriptase polymerase chain reaction for DNA repair enzymes

**Figure 4:**
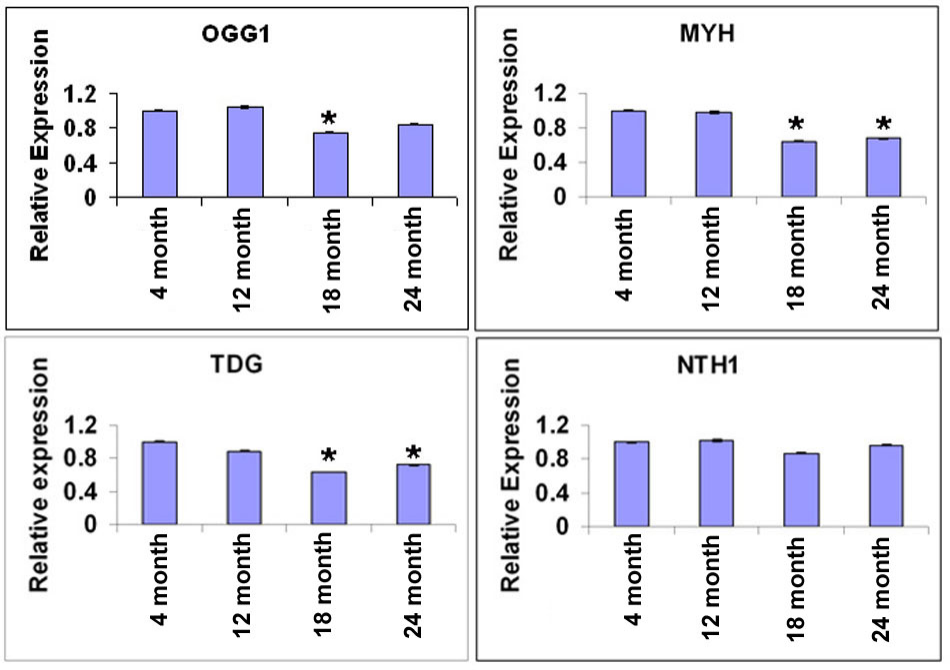
mRNA levels of DNA repair enzymes are decreased in aged retinal pigment epithelium and choroid. Comparisons of the mRNA levels of DNA repair enzymes: OGG1 (**A**), MYH (**B**), TDG (**C**) and NTH1 (**D**), in mouse retinal pigment epithelium (RPE) and choroid at different ages 18s mRNA was used as internal standard for normalization. The expression levels of OGG1 were decreased at 18 month (p=0.01, n=6), but showed no difference at 12 month (p=0.73, n=6) and at 24 month (p=0.07, n=6) as compared with the 4 month group (n=5) (**A**). The expression levels of MYH were decreased at 18 month (p=0.001, n=6) and at 24 month (p=0.007, n=6), but showed no difference at 12 month (p=0.87, n=6), compared with the 4 month group (**B**). The expression levels of TDG were decreased at 18 month (p<0.0001, n=6) and at 24 month (p=0.03, n=6), but showed no difference at 12 month (p=0.35, n=6), compared with the 4 months group (**C**). The expression levels of NTH1 were not different at all ages tested (p>0.05, n=6; Panel **D**). Values are the mean ±SEM.

We investigated the changes in expression of a subset of enzymes associated with DNA repair functions in the mouse RPE and choroid. OGG1, MYH, thymine DNA glycosylase (TDG), and NTH1 remove modified bases in the first step of the DNA repair process [4,7]. Another commonly studied DNA repair enzyme is NTH1. Figure 4 illustrates the results of quantitative RT–PCR experiments for these enzymes comparing RPE and choroid at different ages. The expression level of OGG1 was decreased at 18 month (p=0.01, n=6), but showed no difference at 12 month (p=0.7, n=6) and 24 month (p=0.07, n=6), compared with the 4 month group (Figure 4A). The expression level of MYH was decreased at 18 month (p=0.001, n=6) and 24 month (p=0.007, n=6), but showed no difference at 12 month (p=0.87, n=6), compared with the 4 month group (Figure 4B). The expression levels of TDG was decreased at 18 month (p<0.0001, n=6) and at 24 month (p=0.03, n=6), but showed no difference at 12 month (p=0.35, n=6), compared with the 4 month group (Figure 4C). The expression level of NTH1 was not different at all ages tested (p>0.05, n=6; see Figure 4D).

### Protein levels of DNA repair enzymes

**Figure 5:**
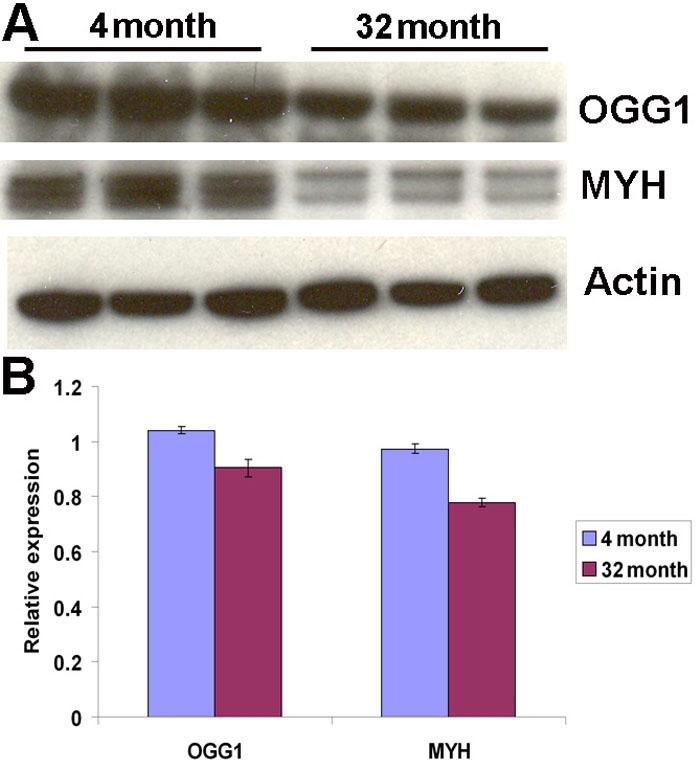
Protein levels of DNA repair enzymes are decreased in aged retinal pigment epithelium and choroid. **A**: Immunoblots of the DNA repair enzymes, OGG1 and MYH, are shown. Actin was used as internal reference. Each group of three bands represents protein extracts from both retinal pigment epithelium (RPE) and choroid of three different animals. **B**: The differences in expression levels were determined by scanning gels and determining the integrated areas of the bands using Image-J software. Data are expressed as normalized ratios to actin. There were significant decreases in aged RPE and choroid of OGG1 (p=0.02, n=3) and MYH (p=0.001, n=3), compared to young RPE and choroid.

Protein levels of the enzymes for DNA repair in RPE and choroid were determined using antibodies against OGG1 and MYH. As shown in Figure 5, there was significant decrease in OGG1 at 32 month (0.91±0.03) (p=0.02, n=3), compared with the 4 month group (1.04±0.01); in MYH at 32 month (0.78±0.01) (p=0.001, n=3), compared with the 4 month group (0.97±0.02).

## Discussion

Our study demonstrates that in the RPE and choroid of retinas from old rodents, there is increased oxidative DNA damage, which is primarily in the mtDNA and is likely associated with age-related deficiencies of DNA repair enzymes. mtDNA is particularly prone to oxidative damage compared to nDNA, because mtDNA is in the matrix of the mitochondria and thus in close proximity to the ROS-generating respiratory chain. In addition, the mitochondrial genome is not covered by histones and other DNA-associated proteins, allowing direct exposure to ROS. Furthermore, mtDNA is an intron-less DNA with a high transcription rate, thus providing a high probability of oxidative modification of the coding region.

In our studies, the oxidative DNA damage marker, 8-OHdG, was increased in RPE and choroid from old rodents. Conversely, expression levels of the DNA repair enzymes were decreased in RPE and choroid from old rodents. Our findings strongly suggest an association between increased oxidative mtDNA damage and decreased DNA repair enzyme capabilities in RPE and choroid as a function of normal aging.

AMD is a degenerative condition of the central, cone-rich zone of the outer retina. Whereas the cause of AMD is not known, multiple factors may be involved, including environment, nutrition, and genes. In AMD, the loss of vision is due to the degeneration of photoreceptors, the specialized neurons in the retina that receive and transduce light into an electrical signal. However, several lines of evidence suggest that dysfunction of the RPE, the cells that support the photoreceptors, is a crucial, early event in the pathogenesis of AMD. The RPE fulfills metabolic functions that are essential for proper activity and survival of retinal photoreceptors. The RPE, which is adjacent to the photoreceptors and rests on Bruch’s membrane, phagocytoses and digests the distal parts of the outer segments of the photoreceptors. Impaired function of the RPE may play an essential role in the initiation of pathophysiological events and subsequent degeneration of the corresponding photoreceptor cells causing AMD. Thus, the RPE may be a key link to the initiation and progression of disease.

Oxidative damage to the macula has been suggested to underlie the development of AMD [15]. Barron et al. [16] showed mtDNA deletions accumulate in the aging photoreceptors of the macula. In our laboratory, we have demonstrated increased damage to mtDNA in the inner segments of the mouse photoreceptors with age (unpublished data). Based on in vitro data from an RPE cell line, the accumulation of mtDNA damage has been suggested as a causative agent or as a susceptibility factor in AMD [11]. Our results are the first to demonstrate that there is increased damage to mtDNA with age in the RPE and choroid in vivo and to suggest that there may be associated decreases in specific DNA repair enzymes in this aged tissue. Our data are consistent with the development of mitochondrial alterations in the RPE in humans with AMD [17]. Thus, the accumulation of damaged mtDNA in the RPE and choroid may be an age-related susceptibility factor for AMD.

Imam et al. showed that there is a differential pattern of loss of DNA repair capability in various CNS tissues [17]. The efficiency of repair of damaged DNA depends on the activities of many DNA repair enzymes [18]. Repair of oxidative DNA damage is mediated by both base excision repair and nucleotide excision repair systems [19]. Repair of DNA bases damaged by oxidation occurs primarily via the DNA base excision repair pathway [20].

Our experiments demonstrate that the RPE and choroid from old rodents have decreased gene expression of key DNA repair enzymes and may therefore have relatively less DNA repair capability. In particular, we found age-related decreases in expression of DNA repair enzymes, such as OGG1, MYH, and TDG that remove modified bases in the first step of the DNA repair process. The decrease in mRNA levels of selected DNA repair enzymes with age may be due to a change in transcription or mRNA stability. Nevertheless, the decrease in cellular protein levels of at least two of these enzymes is likely to lead to decreased repair capacity. Thus, our findings suggest that accumulated oxidative mtDNA damage is a downstream effect of the age-related decreased expression of DNA repair enzymes.

In summary, our data demonstrate that decreased expression of DNA repair enzymes and increased oxidative damage to mtDNA occur in the normal aging process of the RPE and choroid. The decreased expression of DNA repair enzymes in RPE and choroid with age may lead to dysfunction of these tissues and, therefore, susceptibility to AMD. Enhancement of DNA repair mechanisms may provide a strategy to prevent or slow the progress of AMD.
